# A Review of 2011 for *PLoS Computational Biology*

**DOI:** 10.1371/journal.pcbi.1002387

**Published:** 2012-01-26

**Authors:** Rosemary Dickin, Chris James Hall, Laura K. Taylor, Andrew M. Collings, Ruth Nussinov, Philip E. Bourne

**Affiliations:** 1 Public Library of Science, Cambridge, United Kingdom; 2 National Cancer Institute, SAIC-Frederick, Maryland, United States of America; 3 Tel Aviv University, Tel Aviv, Israel; 4 Department of Pharmacology, University of California San Diego, La Jolla, California, United States of America; 5 Skaggs School of Pharmacy and Pharmaceutical Sciences, University of California San Diego, La Jolla, California, United States of America

In 2011, during discussions at various conferences, as well as informally with authors, readers, reviewers, and editors, we were struck by one resonating theme: the view that *PLoS Computational Biology* has helped to create a sense of community amongst a broad group of scientists and educators. While the journal labels itself as a PLoS “community” journal, if that label has any true meaning, it must come from the community itself. We feel that, after six years, we are indeed serving the community well, but as always you can disagree at any time, either publicly with a comment in response to this article or by email (ploscompbiol@plos.org).

That service comes first and foremost from the research we publish, but also from our desire to educate, report on open-source software, provide a history of the field, capture the vision of our editors, and move beyond the boundaries of traditional publishing to inform people within and outside of our community. Before we take a look at developments in each of these areas, and what is to come in 2012, let us first review how we served the community in 2011.

According to Google Analytics, 2011 saw over 553,000 unique visitors to our website and more than two million article views (not including access statistics from PubMed Central). Visitors came from 211 countries/territories, which was undoubtedly helped by the fact that the journal is open access. India, Spain, Russia, and Iran each showed over a 40% increase in visitors from the previous year. From the journal website, the most accessed Research Article was “Effect of Promoter Architecture on the Cell-to-Cell Variability in Gene Expression” by Sanchez et al. [1], published in March 2011 (8,954 views at the time of writing); the most accessed article overall was “Ten Simple Rules for Building and Maintaining a Scientific Reputation” by Bourne and Barbour [2], published in June 2011 (15,255 views at the time of writing).

Also in 2011, 1,623 research articles from 57 countries were submitted, up 16% from 2010, and 384 were published (down 2% from 2010). Receiving more but publishing about the same number in real terms should reflect the increasing quality of our content. We are very grateful to our Associate Editors, Guest Editors, reviewers (a list of Guest Editors and reviewers from 2011 is available in Table S1), and, of course, our Deputy Editors – Patricia Babbitt, Joel Bader, Sebastian Bonhoeffer, Lyle J. Graham, Konrad Kording, Douglas Lauffenburger, Uwe Ohler, Nathan Price, Burkhard Rost, Olaf Sporns, Wyeth Wasserman, and Weixiong Zhang – for helping us to handle this growth. With this growth, we have not met our goal of reducing the times to first decision, even with the addition of new editors, but we will continue to work on this in 2012. Our median decision before review time in 2011 was 8 days, and our median decision after review time was 47 days.

A number of our Research Articles were featured in blogs and the popular press. Notably, Mitra Hartman's paper on the morphology of the rat vibrissal array [3] was covered extensively, including two videos, by National Public Radio and Science Bytes.

Our Software section was launched in August 2011, and we have so far published one article, with six more either accepted or under review. Uptake has been relatively slow, based on, we believe, the open source and stringent documentation requirements we have imposed. We believe it is better to publish only a few, but high-quality, software articles, and that this will highlight the lack of rigor of software otherwise in the field.

Our Education section has continued to flourish, in part because of the journal's relationship with the International Society for Computational Biology (ISCB). This year we introduced a collection, Bioinformatics: Starting Early, which takes the notion of biology as a computational science into secondary schools. We are hoping for more articles from those involved in secondary teaching in 2012. Open science removes all boundaries not only to reading the latest science, but also to contributing to that science. We have even seen secondary school students as authors and expect to see more in the future.

In July 2011 we began the Editors Outlook series, with five published [4]–[8] and more on the way. These mini-reviews already broach subjects from ontologies to genome organization, and from evolution to data and privacy. They speak to the breadth of our field and editorial board, and collectively will form a vision from our many expert editors of what is being, and will be, accomplished in the coming years.

That our journal is fully open access provides opportunities for maximizing the use and reuse of our scholarship; we intend to explore this further in 2012. Early in 2012 we will launch our first Topic Page on circular permutations in proteins. Wikipedia is a valuable resource for knowledge dissemination, yet Wikipedia pages are lacking in coverage of computational biology. In part this is because authors gain little career-based reward for creating Wikipedia pages. We aim to bridge the gap. Topic Page articles, which will be published in the journal and will each receive a PubMed identifier and DOI, will become the copy of record, thereby crediting the author(s). At the same time the Topic Page will be used to seed a Wikipedia article and become a living version of the same material–a viable option thanks to our Creative Commons license. Look for an announcement of this development in the new year, but in the interim if you have ideas for Topic Pages you would like to contribute, please do get in touch for further information (ploscompbiol@plos.org).

We are also contemplating a new article type: Data Pages. Data Pages would be brief publications about datasets, in which the data are not already well described in other papers yet are considered of great value to the community. Such brief publications would bring a traditional reward to the producers of these shared datasets. Which is more valuable: a dataset downloaded and used by 100 investigators, who in turn publish research based on these data, or a paper that is cited only by the authors who wrote it? Data Pages would, from our point of view, help to answer this question.

If you want to provide feedback on our plans for Data Pages later in 2012, please do so by commenting on this article. Feel free to comment in public or to us privately on anything we are doing, or ideas that you have for the future of the journal. After all, *PLoS Computational Biology* is a community journal, and if you have read this far, you should consider yourself an important part of our ever-broadening community.

## Supporting Information

Table S1 Guest Editors and reviewers for *PLoS Computational Biology* in 2011.
(XLS)
